# Personalized setting of plan parameters using feasibility dose volume histogram for auto‐planning in Pinnacle system

**DOI:** 10.1002/acm2.12897

**Published:** 2020-05-04

**Authors:** Wenlong Xia, Fei Han, Jiayun Chen, Junjie Miao, Jianrong Dai

**Affiliations:** ^1^ Department of Radiation Oncology National Cancer Center/National Clinical Research Center for Cancer/Cancer Hospital Chinese Academy of Medical Sciences and Peking Union Medical College Beijing 100021 China

**Keywords:** Auto‐Planning, lung cancer, OAR sparing, planning time, plan quality, VMAT

## Abstract

**Purpose:**

The personalized setting of plan parameters in the Auto‐Planning module of the Pinnacle treatment planning system (TPS) using the PlanIQ feasibility tool was evaluated for lung cancer conventional fractionated radiotherapy (CFRT).

**Materials and method:**

We reviewed the records of ten patients with lung cancer who were treated with volumetric modulated arc therapy (VMAT). Three plans were designed for each patient: the clinically accepted manual plan (MP) and two automatic plans including one generated using the generic plan parameters in technique script (AP1) and the other generated using personalized plan parameters derived based on feasibility dose volume histogram (FDVH) in PlanIQ (AP2). The plans were assessed according to the dosimetric parameters, monitor units, and planning time. A plan quality metric (PQM) was defined according to the clinical requirements for plan assessment.

**Results:**

AP2 achieved better lung sparing than AP1 and MP. The PQM value of AP2 (52.5 ± 14.3) was higher than those of AP1 (49.2 ± 16.2) and MP (44.8 ± 16.9) with *P* < 0.05. The monitor units of AP2 (585.9 ± 142.9 MU) was higher than that of AP1 (511.1 ± 136.5 MU) and lower than that of MP (632.8 ± 143.8 MU) with p < 0.05. The planning time of AP2 (33.2 ± 4.8 min) was slightly higher than that of AP1 (28.2 ± 4.0 min) and substantially lower than that of MP (72.9 ± 28.5 min) with *P* < 0.05.

**Conclusions:**

The Auto‐Planning module of the Pinnacle system using personalized plan parameters suggested by the PlanIQ Feasibility tool provides superior quality for lung cancer plans, especially in terms of lung sparing. The time consumption of Auto‐Planning was slightly higher with the personalized parameters compared to that with the generic parameters, but significantly lower than that for the manual plan.

## 1 INTRODUCTION

Along with software advancements to handle increasingly complex calculations, radiation treatment planning is rapidly becoming more and more automated to relieve the planner of tasks that can be readily handled by computers, such as autosegmentation and optimization.^1^ After localization images of the target are obtained and exported to a treatment planning system (TPS), most of the steps involved in generating the anatomical structure and optimizing the treatment plan can be automated. During the optimization process, the use of automatic planning can reduce human variability while maintaining similar treatment plan quality.

The quality of a manual radiotherapy treatment plan strongly depends on the planner’s experience and the planning time^2^ and, consequently, has large uncertainty. In inverse treatment planning (IMRT/VMAT), the optimization process can be very time consuming because it requires a process of repeated trial‐and‐error. Automatic planning is a solution for standardizing plans, improving the plan quality and decreasing the planning time. There are several commercial tools available for plan automation. The Auto‐Planning (AP) module of Pinnacle (Philips Medical System, Fitchburg, WI) is a software application that simplifies the planning process by using technique scripts and automatic optimization tuning methods. Varian’s RapidPlan (Varian Medical Systems, Palo Alto, CA, USA) uses a database of previously used treatment plans to conduct (knowledge‐based planning). Zhang et al.^3^ implemented a plug‐in (mdaccAutoPlan) in Pinnacle TPS for various tumor sites. Erasmus‐iCycle develops plans based on multicriteria optimization (MCO).^4^ Some studies imply that planning automation can also be useful for adaptive radiation therapy^5^ and unbiased comparisons of treatment techniques.^6^, ^7^, ^8^

The AP module has already been tested in developing plans for head and neck treatments,^9^, ^10^, ^11^, ^12^ hippocampal avoidance whole‐brain treatment,^13^ prostate treatments,^14^ and liver tumor stereotactic body radiotherapy (SBRT).^15^ The results of these studies show that this approach may improve the efficiency of the optimization process, eliminate the need for repeated trial‐and‐error during the manual planning process, and tend to improve the standardized plan quality. In the AP module, it is still necessary to manually set the beam angles, prescription, and the initial optimization parameters in advance. Generally, the selected initial optimization parameters directly affect the final plan quality. For cases with the same clinical requirements, the AP module uses generic initial parameters in the technique script according to in‐house planning experience. However, these generic initial parameters are not appropriate for some cases, necessitating manual modification via trial‐and‐error. PlanIQ Feasibility (Sun Nuclear Corp, Melbourne, FL) is a tool to estimate the best possible sparing dose of organs at risk (OARs) *a priori* before starting the plan optimization.^16^ Assuming an ideal fall‐off from the prescription dose at the target boundary, a feasibility dose volume histogram (FDVH) quantitatively determines the regions of a DVH that are impossible (red), difficult (orange), challenging (yellow), and probable (green) for each organ at risk (OAR). The FDVH allows the OAR planning goals to be personalized according to the patient geometry. In the latest version of Pinnacle v16.2, the PlanIQ Feasibility tool has been integrated into the AP module.

In this study, we evaluate an AP workflow in which the plan parameters are personalized using the PlanIQ Feasibility tool, and verify its effectiveness in developing VMAT plans for 10 lung cancer patients. For each case, three plans were developed: a clinically accepted manual plan (MP), an automatic plan generated using generic initial parameters (AP1), and another automatic plan generated using personalized OAR sparing goals according to the FDVH derived using PlanIQ software (AP2). The treatment plans were assessed comprehensively in terms of a new plan quality metric (PQM), which was defined according to the existing PQM^17^ and quality score, S_D_.^18^

## 2 MATERIALS AND METHODS

### 2.A Patient plan selection and planning objectives

Treatment plans of 10 lung cancer patients using conventionally fractionated radiotherapy (CFRT) were selected from recent patient treatment plans. Computer tomography (CT) scans were captured during normal breathing in the supine position with a slice thickness of 5 mm and plane voxel size of 1 mm × 1 mm. The gross tumor volume (GTV), clinical target volume (CTV), and planning target volume (PTV) were contoured by qualified radiation oncologists. Relevant OARs were also delineated, which mainly included the whole lung, spinal cord, and heart. An extra 5 mm margin was added to the spinal cord as the planning organ‐at‐risk volume (PRV). The tumor staging was listed in Table 1. The prescribed dose was 60 Gy, delivered in 30 fractions.

**Table 1:** Tumor staging.

Tumor staging	Number of patients
T4	3 (N2 = 2/N3 = 1)
T3	1 (N0 = 1)
T2	2 (N2 = 1/N3 = 1)
T1	4 (N0 = 1/N2 = 2/N3 = 1)

The planning objectives for the PTV were that the relative volume that receives ≥ 100% of the prescribed dose > 95%, and the maximum point dose < 110% of the prescribed dose. The dose coverage and homogeneity of the PTV were assessed based on the dose distribution, the dose volume histogram (DVH), and the trade‐off between the dose delivered to the PTV and OAR sparing. The planning objectives for the OARs were as follows: point dose, spinal cord < 40 Gy; point dose, spinal cord PRV < 45 Gy; volume of whole lung receiving more than 5 Gy (V5) not specified though lower doses are preferred, and that receiving more than 20 Gy (V20) < 28% and that receiving more than 30 Gy (V30) <20%; mean dose, whole lung (D_mean_) <17 Gy; and volume of heart receiving more than 30 Gy (V30) < 40% and that receiving more than 40 Gy (V40) < 30%. The constraints specified in our department were mainly based on the quantitative analysis of normal tissue effects in the clinic (QUANTEC) guidelines,^19^, ^20^, ^21^ but were more stringent.

### 2.B Planning process

During the planning process, the arrangement of beams, dose prescription for the PTV, and initial optimization parameters of each OAR were set by loading a predefined technique script. The workflow of technique script was as follows: (a) two arcs including a clockwise (CW) arc and a counterclockwise (CCW) arc were created, ranging from 181° to 30° for tumors located in the right lung and from 330° to 180° for tumors located in the left lung, and the collimator angle of each beam was set according to the target shape from the beam's eye view (BEV). In the technique script, we used Varian Novalis Tx equipped with a 120 multi‐leaf collimator (MLC) (field size: 40 × 40 cm, 5 mm leaf width in central 20 cm of the field, 10 mm leaf width in the outer 20 cm of the field). The settings were as follows: the beam energy was 6 MV, the control point spacing was 4º, and the leaf motion was constrained to 0.5 cm/°. (b) The prescribed dose was defined as 60 Gy, delivered in 30 fractions. (c) The initial settings for the planning objectives were defined according to the clinical requirements. Finally, the AP engine attempted to meet the objectives of target while lowering the dose to the OARs with minimal compromise to the PTV coverage by an optimization process involving six iterative loops and automatic creation of objectives on additional structures. In each progressive loop, target, OARs, and hot/cold spot objectives are added, fine‐tuned with one another, and optimized. The optimizer continues working after the clinical objectives of maximizing target coverage and sparing OARs are met.

Each case includes one manual plan and two automatic plans. The MPs were generated by expert medical physicists with at least 3 yr experience in clinical IMRT/VMAT treatment planning and approved by physicians. The two types of APs were generated by the AP module, one using generic protocol parameters as inputs (AP1) and the other using personalized OAR sparing goals according to the FDVH derived by PlanIQ (AP2). In the Pinnacle v16.2 used in our department, the PlanIQ Feasibility tool was not fully integrated into the TPS at the time of the study. The two software were installed on separated servers with different operating systems (Pinnacle in Unix and PlanIQ in Windows).

**Fig. 1:**
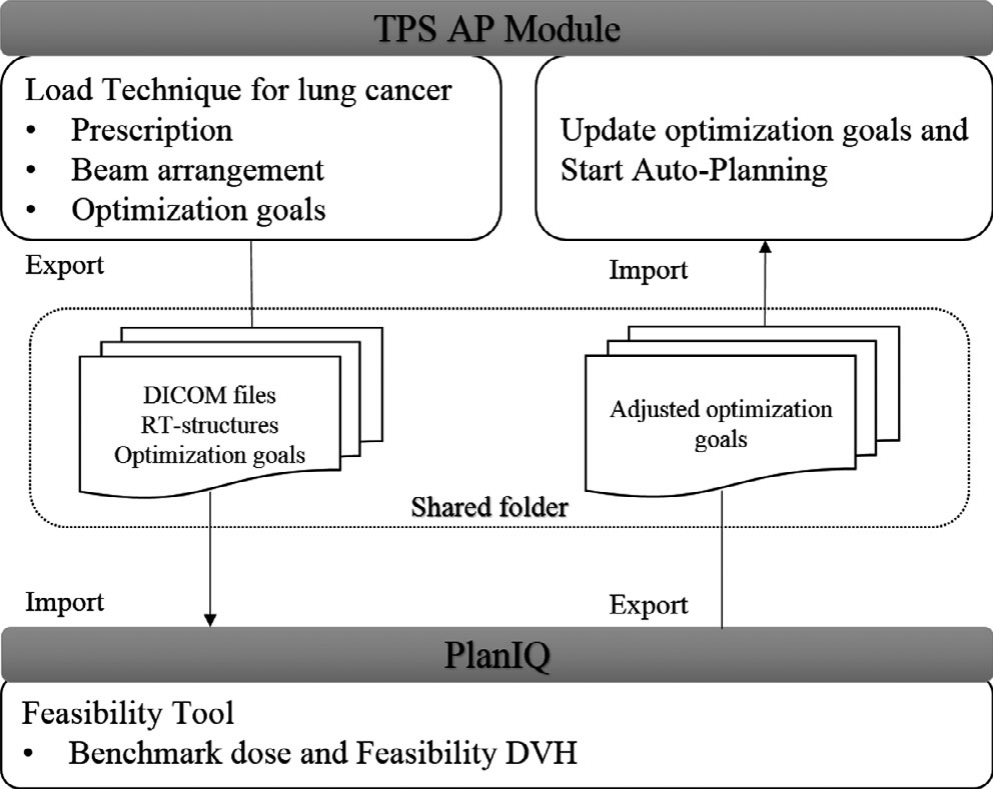
Schematic of the workflow for AP2.

A workflow was developed for the AP2, as shown in Fig. 1. The process involved (a) loading technique script in TPS AP module, (b) exporting the digital imaging and communications in medicine (DICOM) files, radiotherapy (RT) structures, and optimization goals to a shared folder in the Unix system, (c) importing the files to PlanIQ and generating the FDVH; (d) exporting the adjusted optimization goals to the shared folder, and (e) importing the adjusted optimization goals, updating the initial optimization parameters, and starting the AP process.

In the Feasibility tool, the adjusted optimization goals for the lungs were between difficult (orange) and challenging (yellow), and those for the heart and spinal cord were between challenging (yellow) and probable (green). In the AP module, the OAR priority parameters were set as follows (lung: high or medium, compromise; heart: low, compromise; spinal cord: high, no compromise).

### 2.C Study endpoints

The three plans for each patient were compared in terms of the dosimetric parameters: PQM value, total monitor units, and planning time.

The dosimetric parameters are as follows: (a) The homogeneity index (HI) of the PTV was defined as follows:

(1) $$HI=100\%\times \frac{D2\%-D98\%}{D50\%},$$

where D2%, D50%, and D98% are the minimum doses delivered to 2%, 50%, and 98% of the PTV, respectively.^22^ HI closer to 0 indicates better homogeneity in the PTV. (b) The conformity index (CI) of the PTV is defined as follow:

(2) $$CI=\frac{V_{\mathrm{PTV}}\times TV}{TV_{\mathrm{PTV}}^{2}},$$

where V_PTV_ is the volume of the PTV, TV_PTV_ is the portion of the V_PTV_ that is within the prescribed isodose line, and the TV is the treated volume of the prescribed isodose line.^23^ CI closer to 1 indicate better conformity in the PTV. (c) The dose deposition in the lungs was analyzed in terms of the V5 Gy (%), V20 Gy (%), V30 Gy (%), and mean dose (D_mean_). (d) The dose deposition in the heart was analyzed using V30 Gy (%) and V40 Gy (%). (e) The maximum dose (D_max_) to the spinal cord and spinal cord PRV.

As shown in Table 2, a PQM scoring procedure with 10 related submetrics was defined for a treatment plan. Each metric is calculated using a unique quantity and PQM value function, and the ranges of the corresponding PQM values were uniformly set from 0 to 10. The quality score, S, of each plan was defined as the sum of PQM values of the subcomponents,^17^, ^18^ as follows:

(3) $$S=\sum_{i=1}^{k}S_{i},$$

(4) $$S_{i}=\left\{\begin{array}{c} \frac{M_{i}-M_{\mathrm{il}}}{M_{\mathrm{iu}}-M_{\mathrm{il}}}\times S_{i\max},\text{for CI}\\ \frac{M_{\mathrm{iu}}-M_{i}}{M_{\mathrm{iu}}-M_{\mathrm{il}}}\times S_{i\max}\text{,else} \end{array}\right.,$$

where k is the number of subcomponents, S_i_ is PQM value of the metric corresponding to M_i_, S_imax_ is the maximum PQM value (i.e., the highest score) of M_i_, and M_il_ and M_iu_ are the lower and upper limits of M_i_, respectively. The interval for M_i_ was determined based on recorded data of M_i_ for all ten patients. Thus, the lung cancer plans of all patients in this control experiment could be evaluated using this PQM scoring procedure.

**Table 2:** Evaluation interval of metric parameters along with their value range

Structure	Metric				PQM value range	
	Parameter	Lower limit	Interval	Upper limit	Minimum	Maximum
PTV	CI	1	1–2	2	0	10
	HI	0	0–0.2	0.2	0	10
Lungs	V5 (%)	27	27–65	65	0	10
Lungs	V20 (%)	14	14–28	28	0	10
Lungs	V30 (%)	12	12–20	20	0	10
Lungs	Dmean (Gy)	9	9–17	17	0	10
Heart	V30 (%)	3	3–40	40	0	10
Heart	V40 (%)	2	2–30	30	0	10
Spinal cord	Dmax (Gy)	31	31–40	40	0	10
Spinal cord PRV	Dmax (Gy)	35	35–45	45	0	10

### 2.D Statistical analysis

Wilcoxon signed rank tests were carried out to compare the MP, AP1, and AP2 for all obtained dosimetric data. The statistical analyses were performed in SPSS v17 (IBM Corp), with significance set at *P* < 0.05.

## 3 RESULTS

**Fig. 2:**
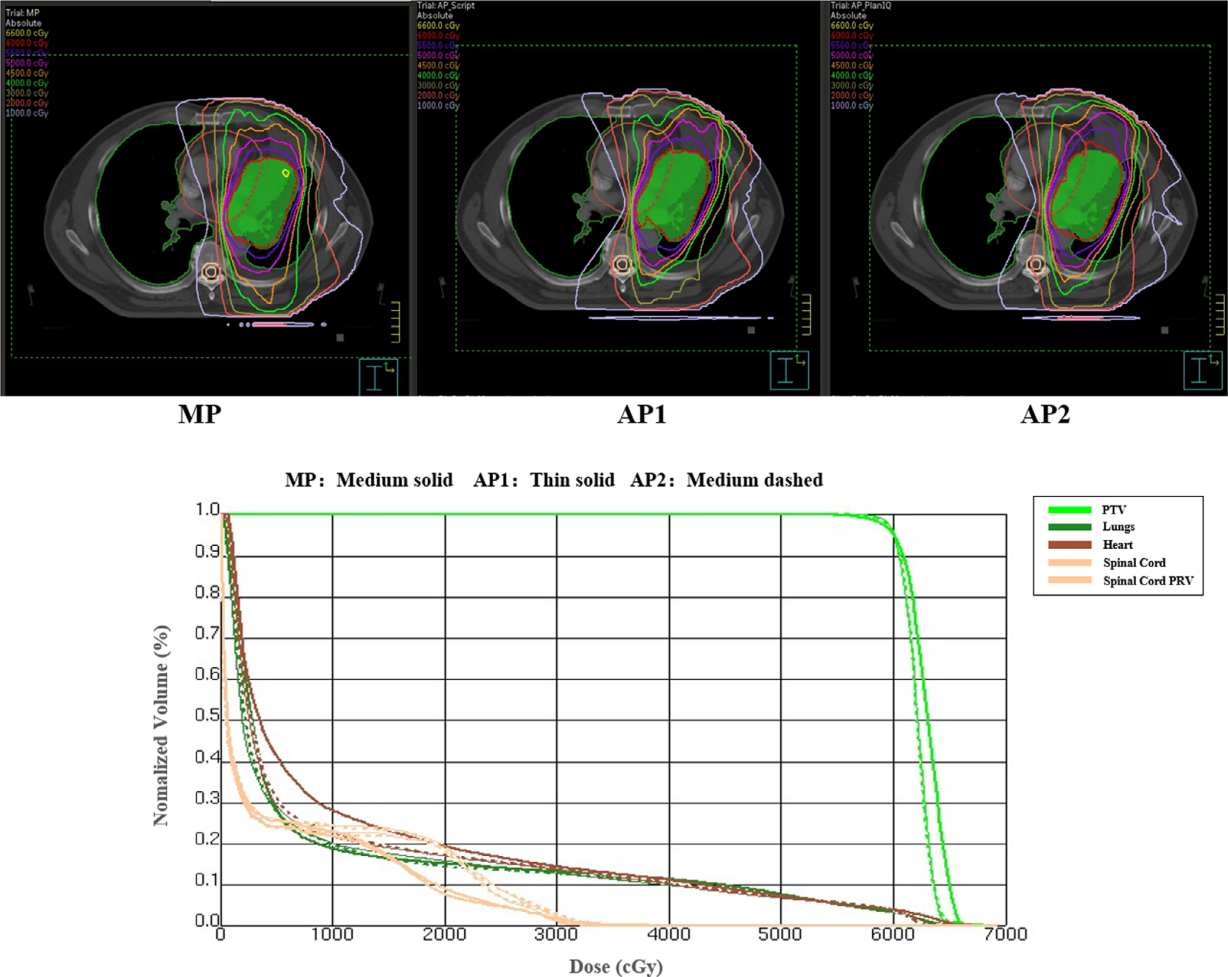
Dose distributions and DVH curves of the three plans.

Figure 2 shows an example of the distribution of the three plans (MP, AP1, and AP2). All three plans satisfied the clinical requirements for all OARs. Table 3 shows the average differences between MP, AP1, and AP2 among all metrics evaluated. There were statistically significant differences for the following parameters: PTV CI, lungs V20 Gy (%), V30 Gy (%), and D_mean_ (Gy); and heart V30 Gy (%), Cord D_max_ (Gy), and Cord PRV D_max_ (Gy). For dose deposition in the lungs, the V20 Gy (%) and V30 Gy (%) of AP2 were lower than those of MP and AP1 (*P* < 0.05), and the D_mean_ (Gy) of AP2 was lower than that of AP1 (*P* < 0.05). For dose deposition in the other OARs, the heart V30 Gy (%), cord D_max_ (Gy), and cord PRV D_max_ (Gy) of AP1 and AP2 were lower than those of MP (*P* < 0.05).

**Table 3:** Averaged differences and 95% confidence interval between each two plans

Type	MP vs. AP1		MP vs. AP2		AP1 vs. AP2	
	Diff (95% confidence interval)	*P*‐values	Diff (95% confidence interval)	*P*‐values	Diff (95% confidence interval)	*P*‐values
PTV						
CI(PTV)	0.00 (−0.10 to 0.10)	0.575	−0.09 (−0.24 to 0.05)	0.241	−0.09 (−0.21 to 0.02)	**0.047**
HI(PTV)	0.01(−0.01 to 0.02)	0.203	0.00 (−0.02 to 0.02)	0.878	−0.01 (−0.02 to 0.00)	0.059
Lungs						
V5 (%)	−1.70 (−5.94 to 2.54)	0.333	−4.49 (−11.86 to 2.87)	0.333	−2.79 (−8.77 to 3.19)	0.285
V20 (%)	−0.58 (−1.39 to 0.24)	0.169	1.62 (0.19 to 3.04)	**0.037**	2.20 (0.94 to 3.45)	**0.007**
V30 (%)	−0.76 (−1.93 to 0.40)	0.114	1.06 (−0.17 to 2.29)	**0.037**	1.82 (1.08 to 2.56)	**0.005**
Dmean (Gy)	−0.26 (−0.61 to 0.08)	0.203	0.30 (−0.08 to 0.68)	0.114	0.56 (0.19 to 0.93)	**0.013**
Heart						
V30 (%)	2.02 (0.42 to 3.63)	**0.028**	2.56 (0.20 to 4.92)	**0.028**	0.54 (−0.74 to 1.82)	0.285
V40 (%)	1.12 (−0.32 to 2.57)	0.139	1.53 (−0.99 to 4.05)	0.203	0.41 (−1.20 to 2.02)	0.799
Spinal cord						
Dmax (Gy)	2.25 (1.21 to 3.28)	**0.013**	2.75 (1.12 to 4.39)	**0.017**	0.50 (−0.33 to 1.34)	0.169
Spinal cord PRV						
Dmax (Gy)	2.54 (1.26 to 3.82)	**0.007**	2.10 (0.33 to 3.88)	**0.047**	−0.44 (−1.61 to 0.73)	0.285

**Fig. 3:**
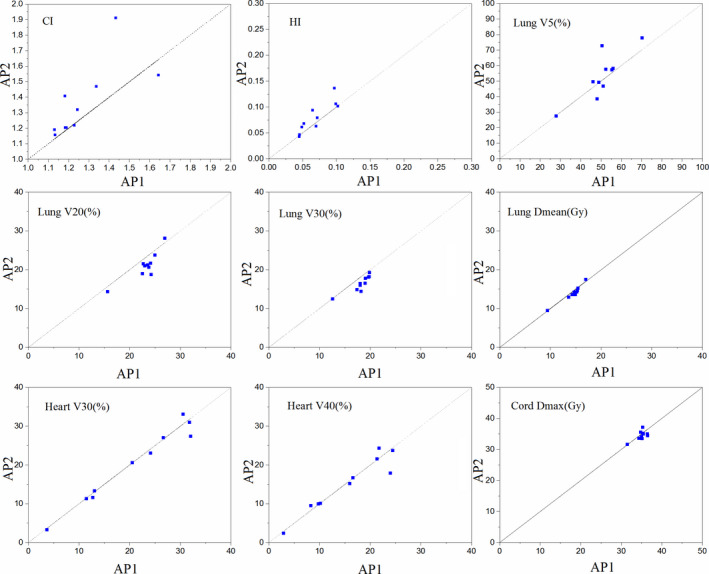
Relationships of the metrics between AP1 and AP2 (n = 10).

Figure 3 shows the relationships of the metrics between AP1 and AP2. The data demonstrate the impact of personalizing the plan parameters compared with traditional AP. Data points for the CI above the dotted line and those for the other parameters below the dotted line indicate that the results of the AP2 were superior.

**Fig. 4:**
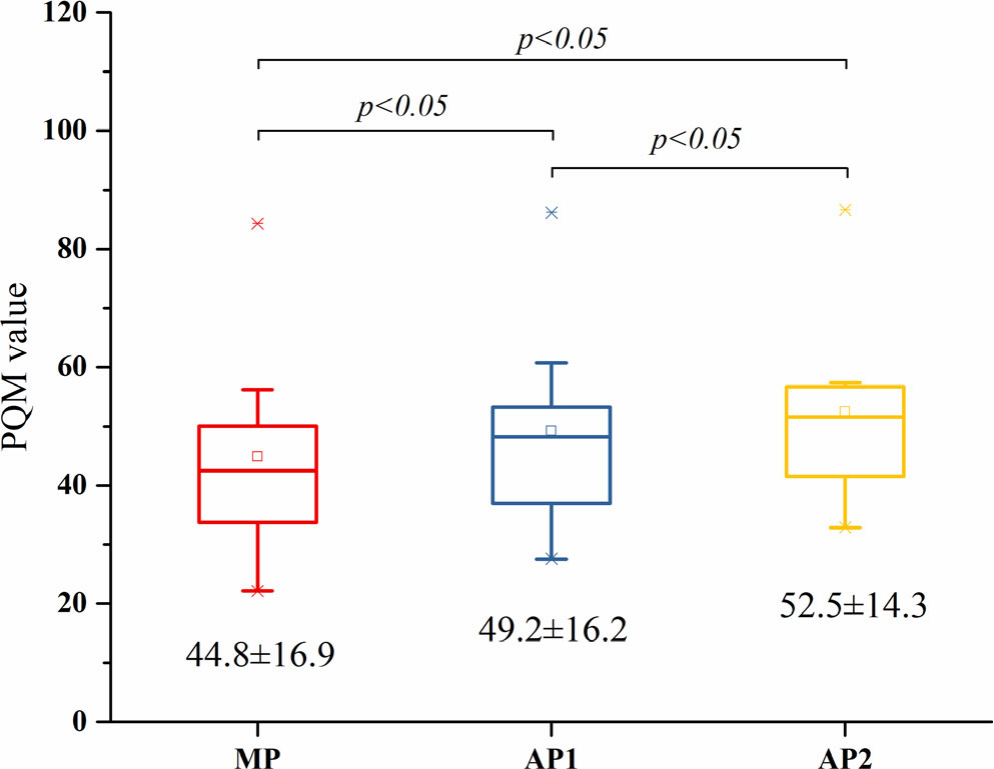
Box‐whisker plots showing the comparison of PQM values.

**Fig. 5:**
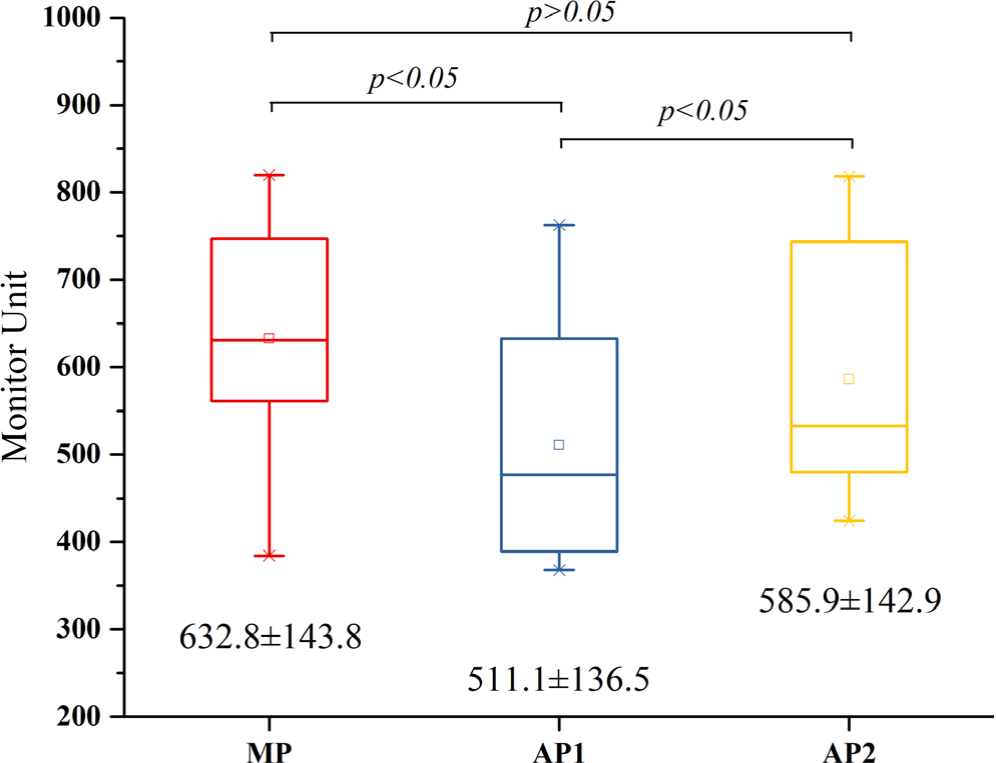
Box‐whisker plots showing the comparison of monitor units.

**Fig. 6:**
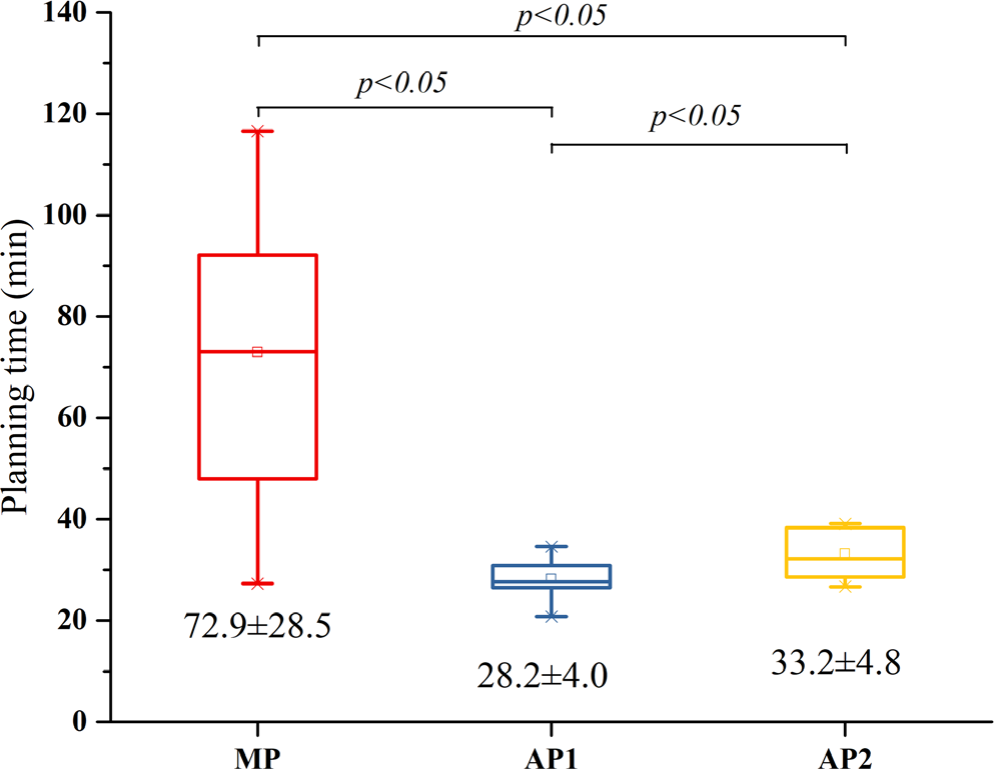
Box‐whisker plots showing the comparison of planning time.

The comparisons of plan quality, monitor units, and planning time are shown from Figs 4, 5, 6 with the corresponding p‐values obtained from the Wilcoxon signed ranks test. The PQM value of AP2 (52.5 ± 14.3) was higher than that of AP1 (49.2 ± 16.2) and MP (44.8 ± 16.9) (*P* < 0.05). The monitor units of AP2 (585.9 ± 142.9) and MP (632.8 ± 143.8) were higher than that of AP1 (511.1 ± 136.5) (*P* < 0.05). The planning times of AP1 (28.2 ± 4.0 min) and AP2 (33.2 ± 4.8 min) were significantly lower than that of MP (72.9 ± 28.5 min) (*P* < 0.05). Since the planning time of AP2 includes the time needed to adjust the optimization goals in PlanIQ (2.3 ± 0.2 min) and the AP time in the AP module (30.9 ± 4.8 min), the planning time of AP2 was higher than that of AP1 (*P* < 0.05).

## 4 DISCUSSION

In this study, we evaluated a technique to personalize plan parameters for VMAT AP in treatment of patients with lung cancer. Three plans (MP, AP1, and AP2) were generated for each case. In AP2, the PlanIQ Feasibility tool was used to generate the personalized plan parameters.

The statistical results in Table 3 show that APs achieved better OAR sparing than the MPs. Furthermore, the dose deposition in the lungs was improved by using personalized parameters; while there was no statistically significant difference between MP and AP1, it appeared that the average lung dose in AP1 was slightly worse than that in MP, even though the priorities of the lung parameters in AP1 were set high. This could be attributed to the use of inappropriate initial settings in AP1 because, although MP and AP1 used the same initial plan parameters, the parameters in MP can be adjusted as needed after the first optimization, while those in AP1 might not be handled well in the following iterations if they are not suitable initially. Therefore, it is beneficial for AP to use personalized initial parameters. Perumal et al.^24^ conducted an evaluation of plan quality improvements in PlanIQ‐guided AP compared to that in radiation therapy oncology group (RTOG)‐guided AP for five cases of different sites, wherein the dose reduction for the mean dose of total lung was 2.2 Gy for single lung cancer case. According to the statistical results of 10 lung cancer cases in this study, significant reduction in lung mean dose of AP2 (0.56 ± 0.52 Gy) was observed (*P* < 0.05). The improvement in plan quality can be directly attributed to the higher degree of personalization of plan parameters.

The relationships of the metrics in Fig. 3 showed that AP2 provided superior protection of the lungs. Although there was no statistically significant difference in the CI and HI of the targets, it still can be observed from Fig. 3 that AP2 had inferior results on CI and HI compared to AP1. This could be attributed to the trade‐off of OAR sparing for AP2. Thus, it is necessary to use a quantitative scoring procedure to assess the overall quality of a treatment plan. The plan quality scoring procedure was defined based on concepts of PQM proposed by Benjamin^17^ and the plan quality score, S_D_, proposed by Bohsung.^18^ The evaluated metrics were the standard clinical OAR constraints for lung cancer treatment, and the score assignments of different metrics were determined by both physicians and planners. It should be noted that differences in PQM values are only meaningful between different plans for one patient. The features of lung cancer plans approved for clinical treatment showed that the HI and CI of the PTV can be properly sacrificed for OARs sparing, especially for decreasing the dose deposition in the lungs.^25^ Thus, the score proportion of the OARs was higher than that of the PTV. However, the scoring procedure may differ between different tumor sites. For example, the radiotherapy plans for nasopharynx cancer (NPC) require better dose coverage and homogeneity of PTV than lung cancer plans, the score proportion of PTV should be increased. According to the plan quality scoring procedure, AP2 provided higher quality than either MP or AP1.

The MP generally has more monitor units than AP1 and AP2, and an average increase of 75 MU of AP2 was observed compared to AP1 in this study. For the ten cases in AP2, the initial optimization parameters of the OARs were generally set with more stringent objectives, and the resulting higher modulation could increase monitor units. Several studies indicated that higher modulation could result in greater segment irregularity and lower pass rate in the dose verification.^26^, ^27^, ^28^ In addition, the AP module significantly reduced the planning time, which is consistent with results reported by Hansen et al.^12^ for head and neck treatments and Gallio et al.^15^ for liver SBRT (both reporting reductions of more than twice). The planning time of AP1 includes loading the technique script and performing AP. The planning time of AP2 includes loading the technique script, obtaining the personalized optimization parameters from PlanIQ, and performing AP. Because the software platforms are separated and additional time is needed for feasibility analysis, AP2 required more planning time than AP1. Since PlanIQ was not fully integrated into the AP module in the Pinnacle v16.2 used in our department, data transmission between the two separated software was required. According to the ten AP2 plans, the average time required for data transmission was approximately 2 min. If the PlanIQ Feasibility tool is fully integrated into Pinnacle and full‐automatic is achieved, the average planning time by AP2 will be further decreased to approximately 31 min. This time‐saving aspect is particularly important for centers with limited resources as it enables planners to focus on more challenging and complex plans.

Generally, the PlanIQ software was used to output plan quality scores according to the relevant metrics and corresponding scoring functions for each target and OAR. With Feasibility tool, *a priori* estimation of the most feasible DVH for OARs can be generated to initialize the optimization goals. Unlike knowledge‐based planning, the Feasibility tool does not depend on prior experience or a learning database of similar treatment plans. The advantages of the method are its simplicity and minimal commissioning effort involved. However, this simplicity leads to limitations. The dose coverage and homogeneity of targets are ideal and unachievable. It approximates the lowest possible boundary of each OAR DVH without consideration of the other OARs. Similar to Pareto optimal, the actual OAR DVH curves can be driven as close as possible to (but never below) the corresponding FDVH, and no individual OAR objective function can be further improved without sacrificing at least one other. The quantitative regions of DVH for each OAR includes impossible (red), difficult (orange), challenging (yellow), and probable (green). Thus, it is not appropriate to put all estimated objectives in difficult regions. Therefore, *a priori* estimated objectives should be chosen from difficult to probable regions based on the priorities of the OARs.

In AP2, the personalized parameters and priorities for the target and OARs were selected by trial‐and‐error. However, this might be a limitation because different initial settings lead to variable plan quality. Although we made an effort to find an appropriate initial setting, this selection may still result in suboptimal plan quality. To address this problem, we can generate several sets of initial parameters within a reasonable scope, and assign different priorities to target coverage and OAR sparing. Then, we can find a “sweet spot” according to these different initial settings. In this way, it is possible and beneficial to further improve the plan quality.

The results of this study indicate that CFRT plans using the AP2 approach are of superior quality to those using AP1 approach or generated manually. In future study, we will continue to test the performance of the AP2 method in SBRT plans. We expect that, by selecting appropriate personalized initial parameters, the AP2 method can achieve better plan quality than the conventional AP1 method. As the automation of planning process continues to improve, it is anticipated that the automatically generated plans will achieve higher quality in a more efficient way; hence, more exploration in this direction is needed.

## 5 CONCLUSIONS

Here, we demonstrated that the use of personalized plan parameters suggested by the PlanIQ Feasibility tool in the AP module of the Pinnacle system provides better quality plans than AP using generic plan parameters. The quality of the plans generated by AP relies on the initial optimization goals, which are generally set by the technique script. By using personalized objectives generated by the PlanIQ Feasibility tool, it is possible to further improve the plan quality and allow planners to use AP in a more effective way. In addition, the planning time of AP was significantly lower than that of MP, and additional time was needed to perform the feasibility analysis for the AP using personalized plan parameters.

## CONFLICTS OF INTEREST

The authors report no conflicts of interest with this study. We declare that we do not have any commercial or associative interest that represents a conflict of interest in connection with this work.

## References

[1] Sharpe MB , Moore KL , Orton CG . Point/counterpoint: within the next ten years treatment planning will become fully automated without the need for human intervention. Med Phys. 2014;41:120601. 25471945 10.1118/1.4894496

[2] Esposito M , Maggi G , Marino C , et al. Multicentre treatment planning inter‐comparison in a national context: the liver stereotactic ablative radiotherapy case. Phys Med. 2016;32:277–283. 26498378 10.1016/j.ejmp.2015.09.009

[3] Zhang X , Li X , Quan EM , Pan X , Li Y . A methodology for automatic intensity‐modulated radiation treatment planning for lung cancer. Phys Med Biol. 2011;56:3873–3893. 21654043 10.1088/0031-9155/56/13/009

[4] Voet PW , Dirkx ML , Breedveld S , Al‐Mamgani A , Incrocci L , Heijmen BJ . Fully automated volumetric modulated arc therapy plan generation for prostate cancer patients. Int J Radiat Oncol Biol Phys. 2014;88:1175–1179. 24529714 10.1016/j.ijrobp.2013.12.046

[5] Li T , Wu Q , Zhang Y , et al. Strategies for automatic online treatment plan reoptimization using clinical treatment planning system: a planning parameters study. Med Phys. 2013;40:111711. 24320419 10.1118/1.4823473

[6] Boylan C , Rowbottom C . A bias‐free, automated planning tool for technique comparison in radiotherapy – application to nasopharyngeal carcinoma treatments. J Appl Clin Med Phys. 2014;15:213–225. 10.1120/jacmp.v15i1.4530 PMC5711248 24423853

[7] Cagni E , Botti A , Micera R , et al. Knowledge‐based treatment planning: an inter‐technique and inter‐system feasibility study for prostate cancer. Phys Med. 2017;36:38–45. 28410684 10.1016/j.ejmp.2017.03.002

[8] Sharfo AW , Voet PW , Breedveld S , Mens JW , Hoogeman MS , Heijmen BJ . Comparison of VMAT and IMRT strategies for cervical cancer patients using automated planning. Radiother Oncol. 2015;114:395–401. 25725503 10.1016/j.radonc.2015.02.006

[9] Hazell I , Bzdusek K , Kumar P , et al. Automatic planning of head and neck treatment plans. J Appl Clin Med Phys. 2016;17:272–282. 26894364 10.1120/jacmp.v17i1.5901 PMC5690191

[10] Gintz D , Latifi K , Caudell J , et al. Initial evaluation of automated treatment planning software. J Appl Clin Med Phys. 2016;17:331–346. 27167292 10.1120/jacmp.v17i3.6167 PMC5690942

[11] Krayenbuehl J , Norton I , Studer G , Guckenberger M . Evaluation of an automated knowledge based treatment planning system for head and neck. Radiat Oncol. 2015;10:226. 26555303 10.1186/s13014-015-0533-2 PMC4641383

[12] Hansen CR , Bertelsen A , Hazell I , et al. Automatic treatment planning improves the clinical quality of head and neck cancer treatment plans. Clin Transl Radiat Oncol. 2016;1:2–8. 29657987 10.1016/j.ctro.2016.08.001 PMC5893480

[13] Wang S , Zheng D , Zhang C , et al. Automatic planning on hippocampal avoidance whole‐brain radiotherapy. Med Dosim. 2017;42:63–68. 28237294 10.1016/j.meddos.2016.12.002

[14] Nawa K , Haga A , Nomoto A , et al. Evaluation of a commercial automatic treatment planning system for prostate cancers. Med Dosim. 2017;42:203–209. 28549556 10.1016/j.meddos.2017.03.004

[15] Gallio E , Giglioli FR , Girardi A , et al. Evaluation of a commercial automatic treatment planning system for liver stereotactic body radiation therapy treatments. Phys Med. 2018;46:153–159. 29519402 10.1016/j.ejmp.2018.01.016

[16] Ahmed S , Nelms B , Gintz D , et al. A method for a priori estimation of best feasible DVH for organs‐at‐risk: validation for head and neck VMAT planning. Med Phys. 2017;44:5486–5497. 28777469 10.1002/mp.12500

[17] Nelms BE , Robinson G , Markham J , et al. Variation in external beam treatment plan quality: an inter‐institutional study of planners and planning systems. Pract Radiat Oncol. 2012;2:296–305. 24674168 10.1016/j.prro.2011.11.012

[18] Bohsung J , Gillis S , Arrans R , et al. IMRT treatment planning:‐ a comparative inter‐system and inter‐centre planning exercise of the ESTRO QUASIMODO group. Radiother Oncol. 2005;76:354–361. 16154218 10.1016/j.radonc.2005.08.003

[19] Kirkpatrick JP , van der Kogel AJ , Schultheiss TE . Radiation dose‐volume effects in the spinal cord. Int J Radiat Oncol Biol Phys. 2010;76:S42–49. 20171517 10.1016/j.ijrobp.2009.04.095

[20] Marks LB , Bentzen SM , Deasy JO , et al. Radiation dose‐volume effects in the lung. Int J Radiat Oncol Biol Phys. 2010;76:S70–S76. 20171521 10.1016/j.ijrobp.2009.06.091 PMC3576042

[21] Gagliardi G , Constine LS , Moiseenko V , et al. Radiation dose‐volume effects in the heart. Int J Radiat Oncol Biol Phys. 2010;76:S77–S85. 20171522 10.1016/j.ijrobp.2009.04.093

[22] Gregoire V , Mackie TR , Neve WD . Prescribing, recording, and reporting photon‐beam intensity‐modulated radiation therapy (IMRT). J ICRU. 2010;10:NP.3‐NP.

[23] Yoo S , Wu QJ , Lee WR , Yin FF . Radiotherapy treatment plans with RapidArc for prostate cancer involving seminal vesicles and lymph nodes. Int J Radiat Oncol Biol Phys. 2010;76:935–942. 20044214 10.1016/j.ijrobp.2009.07.1677

[24] Perumal B , Sundaresan HE , Ranganathan V , Ramar N , Anto GJ , Meher SR . Evaluation of plan quality improvements in PlanIQ‐guided autoplanning. Rep Pract Oncol Radiother. 2019;24:533–543. 31641339 10.1016/j.rpor.2019.08.003 PMC6796777

[25] Miao J , Yan H , Tian Y , et al. Reducing dose to the lungs through loosing target dose homogeneity requirement for radiotherapy of non small cell lung cancer. J Appl Clin Med Phys. 2017;18:169–176. 29024297 10.1002/acm2.12200 PMC5689922

[26] McNiven AL , Sharpe MB , Purdie TG . A new metric for assessing IMRT modulation complexity and plan deliverability. Med Phys. 2010;37:505–515. 20229859 10.1118/1.3276775

[27] Park JM , Park SY , Kim H , Kim JH , Carlson J , Ye SJ . Modulation indices for volumetric modulated arc therapy. Phys Med Biol. 2014;59:7315–7340. 25383976 10.1088/0031-9155/59/23/7315

[28] Crowe SB , Kairn T , Middlebrook N , et al. Examination of the properties of IMRT and VMAT beams and evaluation against pre‐treatment quality assurance results. Phys Med Biol. 2015;60:2587–2601. 25761616 10.1088/0031-9155/60/6/2587

